# Allogeneic hematopoietic stem cell transplantation for mucopolysaccharidosis patients: a single-center experience and assessment of quality of life

**DOI:** 10.1186/s13052-025-01919-7

**Published:** 2025-03-18

**Authors:** Wen Zhang, Yonglan Huang, Xueying Su, Xiaoyuan Zhao, Huiying Sheng, Cuili Liang, Minyan Jiang, Chunhua Zeng, Yanna Cai, Yunting Lin, Yongxian Shao, Sha liu, Hua Jiang, Li Liu

**Affiliations:** 1 Department of Genetics and Endocrinology, Guangzhou Women and Children’s Medical Center, 9 Jinsui Road, Guangzhou, 510623 Guangdong China; 2 Department of Guangzhou Newborn Screening Center, Guangzhou Women and Children’s Medical Center, Guangzhou Medical University, Guangzhou, China; 3 Department of Hematology and Oncology, Guangzhou Women and Children’s Medical Center, Guangzhou Medical University, Guangzhou, China

**Keywords:** Mucopolysaccharidosis, Allogeneic hematopoietic stem cell transplantation, Outcomes, Quality of life

## Abstract

**Background:**

Allogeneic hematopoietic stem cell transplantation (HSCT) has proven to be a viable treatment option for patients with mucopolysaccharidoses (MPS). We investigate the efficacy and improvements in the quality of life of HSCT in pediatric patients with MPS.

**Methods:**

A retrospective analysis of transplantation data from 46 cases of MPS from a single institution in China was conducted.

**Results:**

The cohort of 46 patients included 9 cases of MPS I, 16 cases of MPS II, 15 cases of MPS IVA and 6 cases of MPS VI. The median age at diagnosis was 2.59 years. The median age at transplantation was 3.80 years. The median follow-up time was 3.1 years (range, 0.8–8.1 years) and 43 patients were alive. The incidence of grades II to IV aGVHD was 17.4%, wherein the incidence of grades III and IV aGVHD was 4.3%. The incidence of moderate-to-severe cGVHD was 6.5%. GAGs urinary excretion decreased and enzyme activity levels reached normal. After HSCT, multiple bone dysplasia, upper-airway obstruction and recurrent otitis media were significantly improved; vision, corneal clouding, cardiovascular disease, hepatosplenomegaly and hydrocephalus were improved or remained stable; neurological symptoms were improved or remained stable in most patients but progressed in others; the patients with MPS IH/S and MPS II reached nearly normal growth rate of height and weight. Meanwhile, the patients with MPS IH, MPS IVA and MPS VI remained poor growth after HSCT. The Activities of Daily Living (ADL) scores were improved in most patients with MPS. ADL scores in patients with severe phenotypes were lower than health control subjects and patients with attenuated phenotypes.

**Conclusions:**

HSCT is a good therapeutic option for MPS and improves the quality of life of patients. MPS patients with attenuated phenotypes provide a better outcome in ADL after HSCT.

**Supplementary Information:**

The online version contains supplementary material available at 10.1186/s13052-025-01919-7.

## Background

Mucopolysaccharidosis (MPS) comprises a group of lysosomal storage diseases caused by deficiency of specific lysosomal enzymes [1]. The enzyme deficiency results in disorders in the degradation of glycosaminoglycans (GAGs), with accumulation of undegraded GAGs in the body leading to multi-organ system failure. MPS is classified into 8 types: types І, II, III, IV, VI, VII, IX and X [2]. All types of MPS are autosomal recessive inherited diseases, except for MPS II which is an X-linked inherited disease. MPS X is caused by a deficiency of the enzyme arylsulfatase K (ARSK). Glucuronate-2-O-sulfation occurs in heparan sulfate (HS), dermatan sulfate (DS), and chondroitin sulfate (CS) and, during degradation, is selectively removed by ARSK [2]. Vasilev et al. reported a novel disease of impaired GAGs metabolism without deficiency of known lysosomal enzymes—mucopolysaccharidosis-plus syndrome (MPSPS) [3]. MPSPS, whose pathophysiology is not elucidated, is an autosomal recessive multisystem disorder caused by a specific mutation p.R498W in the VPS33A gene [3].

Currently, Allogeneic hematopoietic stem cell transplantation (HSCT) and enzyme replacement therapy (ERT) are the two main treatments for MPS patients. To date ERT is available for MPS I, MPS II, MPS IVA, MPS VI and MPS VII [4]. ERT can effectively improve some clinical symptoms of patients with each type of MPS [5]. However, the enzyme cannot cross the blood-brain barrier and has little effect on neurological symptoms; moreover, ERT is a long-term and expensive treatment [6]. HSCT is considered the preferred treatment for patients with MPS IH diagnosed before age 2.5 years and an optional treatment for those with MPS IH/S and MPS IS, MPS II, MPS IVA, MPS VI, and MPS VII [7–9]. HSCT is a one-time procedure in which donor stem cells provide the recipient with a continuous source of enzyme [9]. Nonetheless, its use is limited by several factors including the difficulty of finding a matched donor, as well as the transplantation-related morbidity and mortality. With the development of transplantation technology, the mortality rates have declined and engraftment survival rates has improved [8].

Since 2013, 46 MPS cases have received HSCT in our center. This study is the largest single-institution report on HSCT for MPS patients. In this study, we summarize the experience, innovative regimens, the choice of donor, the Activities of Daily Living (ADL) scores and effectiveness of HSCT for each type of MPS.

## Methods

### Patients

A total of 46 patients were diagnosed with MPS at Guangzhou Women and Children’s Medical Center of Guangzhou Medical University. The diagnostic criteria included clinical phenotype and measurement of leukocyte lysosomal enzyme activity: α-L-iduronidase, iduronate-2-sulfatase, galactosamine-6-sulfatase, β-galactosidase, or arylsulfatase B. The concentration of urinary GAGs was measured using the Dimethylene Blue assay normalized to urinary creatinine. Gene mutation analysis was performed by Sanger sequencing for all patients.

From December 2013 to March 2021, we performed HSCT on 46 patients with MPS in our center. Medical history, physical examination, routine laboratory tests, and imaging data were collected. For the 46 patients, the mean follow-up time was 4.5 years, and the median follow-up time was 3.1 years (range, 0.8–8.1 years).

Informed consent was obtained from all patients’ parents. The study was approved by the Institutional Review Board of Guangzhou Women and Children’s Medical Center and all methods were performed in accordance with the relevant guidelines and regulations.

### Eligibility for HSCT

Because of drug accessibility and affordability of enzyme replacement therapy for MPS in China, the majority of patients take HSCT as preferred treatment option. In China, HSCT is the preferred treatment strategy for patients diagnosed before the age of 2.5 years and with presumed severe MPS I (MPS IH). And HSCT can be considered as a therapeutic strategy for patients above 2.5 years of age with MPS IH or patients with MPS IH/S, MPS II, MPS IVA, MPS VI and MPS VII [10].

HSCT recipients acquired donor cells from two different sources: cord blood (CB) and peripheral blood stem cells (PBSCs). For donor hierarchy, a noncarrier HLA-matched related donor, matched unrelated cord blood (UCB), or matched unrelated donor was used. If these donors were not available, an unrelated donor with one to four HLA mismatches or a related donor with haplo-identical matches were considered.

### Conditioning regimen

A busulfan-based myeloablative regimen was applied as the conditioning regimen. Busulfan + cyclophosphamide + thymoglobulin was the conditioning regimen used for PBSC transplantation. Conditioning consisted of administration of intravenous busulfan (4 mg/kg/day, days -9 to -6, total dose 16 mg/kg), cyclophosphamide (50 mg/kg/day, days -5 to -2, total dose 200 mg/kg), and ATG (thymoglobulin, 3.3 mg/kg/day, days -4 to -2, total dose 10 mg/kg; Sanofi, France).

ATG (pre-) + fludarabine + busulfan + cyclophosphamide was the conditioning regimen used for UCB transplantation. Conditioning consisted of administration of intravenous ATG (thymoglobulin, 2 mg/kg/day, days -13 to -11, total dose 6 mg/kg; Sanofi, France), fludarabine (40 mg/m^2^, days -10 to -6, total dose 200 mg/m^2^), busulfan (4 mg/kg/day, days -9 to -6, total dose 16 mg/kg), and cyclophosphamide (50 mg/kg/day, days -5 to -2, total dose 200 mg/kg).

### GVHD prophylaxis

For GVHD prophylaxis, patients received a combination of intravenous cyclosporin (CsA), 2.5 mg/kg/day beginning on day -1 for + 5 days followed by intravenous CsA at 3 mg/kg/day and converted to 12.5 mg/kg/day orally when oral medication could be tolerated. CsA was continued for 100 days and tapered off over 3 months. Intravenous methotrexate (MTX) was administered at 15 mg/m on day + 1 and at 10 mg/m^2^ on days + 3 and + 6. Mycophenolate Mofetil (MMF) was administered at 15∼30 mg/kg/d orally beginning on day -1 for 1 month.

Patients were kept in a sterile isolator with laminar air flow. Standard medication against Pneumocystis carinii, viral, and fungal infections was used. Intravenous immune globulin was administered weekly through to day 100.

### Assessment of HSCT outcome and patient follow-up

Post-transplantation evaluations were performed including symptoms, signs, routine laboratory tests, imaging data, urine GAG assay, enzyme activity assay. Intellectual ability was confirmed by the Gesell Developmental Scale(for children aged under 6 years) or the revised Wechsler Intelligence Scale for Children (for children aged over 6 years) [10]. A successful myeloid engraftment was identified based on achievement of the absolute neutrophil count (ANC) exceeding 0.5 × 10^9^/L for three consecutive days. Chimerism analysis by short tandem repeats (STR)-PCR was performed at 1 month, 3 months, 6 months and 1 year after HSCT and then yearly. Complete chimerism was defined as ≥ 95% donor-derived hematopoietic cells and mixed chimerism was defined as less than 95% donor-derived hematopoietic cells. Acute GVHD and chronic GVHD were defined and graded according to standard criteria [11, 12].

### Questionnaires for activity of daily living (ADL)

The questionnaires for ADL were described previously by Tanjuakio J et al. [13]. The ADL questionnaires were originally developed for patients with MPS II and used in the studies in MPS II and MPS IVA [14]. It assesses movement (walking, movement on stairs, grasping/finger movement, and endurance in a 6 MWT), movement with cognition (toileting, changing clothes, bathing, and eating), cognition ( understanding of everyday conversation, conversation and speaking with others, social participation, and problem solving). Each domain with six subcategories scored from 0 to 5 (0 being the inability to perform a task without maximum assistance and 5 being the ability to perform a task without any assistance). The maximum score was 20 points per domain for a total of 60 points.

Questionnaires were sent to the families, completed directly by the patient and/or the patient’s parent/guardian, and then returned to the study group. Seven of 9 patients with MPS I (1 patient died and 1 was under 5 years old), 12 of 16 patients with MPS II (1 patient died and 3 was under 5 years old), 10 of 15 patients with MPS IVA (1 patient died and 4 was under 5 years old), and 5 patients with MPS VI (1 was under 5 years old) over 5 years of age answered the questionnaires. These 34 patients ranged between 5y2m and 14y4m (mean: 8.46y). ADL questionnaires were obtained from healthy control individuals (5y-15y) after informed consent and then de-identified before inclusion in the study.

### Statistical analysis

Means and standard deviations of the total score and each domain were calculated. Student’s t-test was used to compare MPS patients with age-matched untreated patients and normal controls over 5 years of age. The statistical analysis was performed using SPSS for Windows (version 13.0, SPSS, Chicago, IL, USA). The significance level was set at *p* < 0.05.

## Results

### Patient characteristics

The cohort of 46 patients included 9 cases of MPS I, 16 cases of MPS II, 15 cases of MPS IVA and 6 cases of MPS VI. The first HSCT for MPS in our center was performed in December 2013. There were 33 boys and 13 girls. The median age at diagnosis was 2.59 years (range: 5 m-6y). The median age at transplantation was 3.80 years (range: 9 m-8y11m). Respectively, the median age at transplantation was 4.10 years (range: 9 m-8y11m) for MPS I, 3.91 years (range: 1y1m-6y6m) for MPS II, 3.62 years (range: 1y6m-7y4m) for MPS IVA, and 3.50 years (range: 1y2m-6y8m) for MPS VI (Table 1).

**Table 1:** Patient’s characteristics

Patients no	Sex	Type	Gene	Age at diagnosis	Age at HSCT	Ht-SDS at HSCT	Date of HSCT	Phenotype	Genotype	Current age	Cureent Ht-SDS
1	F	MPS I	IDUA	2y8m	3y9m	-3.4	31/08/2015	Severe	M1T/IVS2-3 C > G	10y2m	-3.6
2	F	MPS I	IDUA	3y3m	3y5m	-1.2	02/02/2018	Intermediate	H33*/L346R	7y5m	-1.2
3	M	MPS I	IDUA	2y7m	5y2m	-3.8	19/06/2018	Severe	V567Sfs/R628*	8y9m	-4.0
4	M	MPS I	IDUA	2y	3y4m	-4.4	08/01/2019	Severe	G220Rfs/Q370*	6y5m	-4.5
5	M	MPS I	IDUA	5 m	1y5m	-1.1	12/07/2019	Severe	E404*/IVS4 + 2 C > T	Deceased	NA
6	F	MPS I	IDUA	2y3m	5y6m	-3.9	09/10/2019	Severe	Q500*/Q500*	7y9m	-4.1
7	F	MPS I	IDUA	2y5m	8y11m	-3.3	18/11/2019	Intermediate	G168V/V304Gfs	11y1m	-3.2
8	M	MPS I	IDUA	6 m	9 m	+ 0.1	26/06/2020	Severe	R89Q/R89Q	2y4m	-4.6
9	M	MPS I	IDUA	3y7m	4y8m	-2.4	10/08/2020	Intermediate	R619G/R619G	6y2m	-3.2
10	M	MPS II	IDS	5y11m	6y	-0.7	09/10/2014	Mild	N350H	13y4m	-2.8
11	M	MPS II	IDS	1y2m	4y9m	-0.5	08/05/2017	Severe	T50Pfs	9y5m	-0.7
12	M	MPS II	IDS	5y4m	6y6m	-0.3	30/09/2017	Severe	R443*	Deceased	NA
13	M	MPS II	IDS	2y8m	2y11m	-0.3	06/11/2017	Mild	G374=	7y2m	-1.6
14	M	MPS II	IDS	2y4m	3y2m	-1.5	26/09/2018	Severe	Recombination	6y6m	-1.8
15	M	MPS II	IDS	7 m	1y1m	+ 0.2	08/11/2018	Mild	T500I	4y3m	-0.3
16	M	MPS II	IDS	1y11m	2y5m	+ 0.1	26/02/2019	Mild	R88L	5y4m	-0.5
17	M	MPS II	IDS	4y	4y6m	+ 0.1	27/05/2019	Mild	L135R	7y3m	-0.8
18	M	MPS II	IDS	1y11m	3y7m	-0.2	20/06/2019	Severe	IVS6 + 1G > C	5y2m	-1.0
19	M	MPS II	IDS	2y7m	3y2m	-0.4	04/11/2019	Severe	IVS4 + 2T > G	5y5m	-0.5
20	M	MPS II	IDS	3y	4y	-0.3	09/01/2020	Severe	IVS6-1G > A	6y1m	-1.0
21	M	MPS II	IDS	2y11m	3y7m	-0,2	05/03/2020	Severe	L359*	5y6m	-0.1
22	M	MPS II	IDS	5y10m	6y5m	-2.1	05/11/2020	Severe	D478N	8y1m	-3.2
23	M	MPS II	IDS	1y9m	2y4y	+ 0.9	03/07/2020	Severe	IVS4 + 1G > A	3y10m	+ 0.7
24	M	MPS II	IDS	3y	3y4m	+ 0.1	09/12/2020	Severe	S117del	4y6m	-0.6
25	M	MPS II	IDS	4y5m	4y9m	-1.1	09/03/2021	Severe	H226D	5y8m	-1.2
26	M	MPS IVA	GALNS	1y10m	3y7m	-3.0	27/03/2014	Severe	M318R/M318R	10y5m	-7.5
27	F	MPS IVA	GALNS	3y5m	7y4m	-5.5	28/08/2015	Severe	P77R/IVS11 + 1G > A	12y11m	-7.4
28	M	MPS IVA	GALNS	1y1m	1y11m	-2.8	08/07/2016	Severe	M318R/M318R	7y6m	-6.0
29	F	MPS IVA	GALNS	1y	2y7m	-3.2	10/10/2016	Severe	D233N/G340D	7y10m	-7.7
30	F	MPS IVA	GALNS	6y	6y11m	-5.2	12/10/2016	Severe	M318R/L440Rfs*54	12y3m	-6.7
31	M	MPS IVA	GALNS	2y	5y8m	-3.1	03/11/2017	Severe	T312A/E477_Q485del	9y11m	-5.2
32	F	MPS IVA	GALNS	3y4m	4y	-3.1	19/12/2017	Severe	F399L	8y2m	-6.8
33	M	MPS IVA	GALNS	3y1m	3y9m	-2.1	08/01/2018	Severe	P81Lfs/P125L	8y1m	-4.9
34	F	MPS IVA	GALNS	2y	2y11m	-2.2	09/01/2018	Severe	Y133C/R386C	7y	-5.9
35	F	MPS IVA	GALNS	4y1m	5y	-4.6	23/09/2019	Severe	M318R/E51*	7y4m	-5.8
36	M	MPS IVA	GALNS	1y1m	1y6m	-3.6	17/02/2020	Severe	G155R/M318R	3y4m	-4.5
37	F	MPS IVA	GALNS	2y2m	2y11m	-2.1	02/03/2020	Severe	P125L/M318R	4y9m	-3.1
38	F	MPS IVA	GALNS	2y6m	3y	-1.6	01/04/2020	Severe	D39Rfs/IVS5-2 A > T	4y10m	-3.3
39	M	MPS IVA	GALNS	1y1m	1y7m	-1.7	24/10/2020	Severe	G139S/*523E	Deceased	NA
40	M	MPS IVA	GALNS	9 m	1y7m	-1.8	18/01/2021	Severe	M318R/P151L	2y8m	-2.5
41	M	MPS VI	ARSB	4y8m	5y5m	-2.2	12/12/2013	Severe	F399L/F399L	13y6m	-3.3
42	F	MPS VI	ARSB	2y5m	6y8m	-5.3	24/05/2014	Severe	Y175D/F399L	14y4m	-7.3
43	M	MPS VI	ARSB	11 m	1y2m	-2.0	20/07/015	Severe	R160*/G328R	7y8m	-5.6
44	M	MPS VI	ARSB	3y5m	3y10m	-3.2	11/12/2015	Severe	R327*/R327*	10y	-5.8
45	M	MPS VI	ARSB	2y	2y5m	-0.8	15/03/2018	Severe	W450C/V358M	6y3m	-1.4
46	M	MPS VI	ARSB	1y2m	1y6m	-1.9	09/10/2020	Severe	F399L/L82P	2y9m	-2.5

The affected 46 patients had GAG in the urine between 16.7 and 221.1 g/mol creatinine, beyond the average reference values for age. The lysosomal enzyme activity analyzed in peripheral blood leukocytes from the 46 patients was significantly lower than the reference range. Sequencing analysis of related genes identified different pathogenic mutations in all 46 patients (Table 1).

### Donor characteristics

For the donor source, non-carrier matched sibling donors were selected in 5 cases (10/10 on high-resolution typing); identical UCB grafts were selected in 18 cases (6/6 on intermediate resolution); identical matched unrelated donors were selected in 9 cases (10/10 on high-resolution typing); unrelated donors with one to two HLA mismatches were selected in 7 cases with added cord blood to minimize the risk of GVHD; mismatched related donors were selected in 7 cases (5/10 on high-resolution typing).

### Transplant-related mortality and complications

The median follow-up time was 3.1 years (range, 0.8–8.1 years), 43 patients (43/46, 93.5%) were alive, well and engrafted. Three patients had died (3/46, 6.5%). Two patients died of severe septicemia, pneumonia and respiratory failure with grades III and IV GVHD (No.5, 12). One died of interstitial pneumonia infection (RSV) at day 30 after transplantation (No.40). The incidence of grades II to IV acute GVHD (aGVHD) was 17.4% (8 of 46), and the incidence of grades III and IV aGVHD was 4.3% (2 of 46). The incidence of moderate-to-severe chronic GVHD was 6.5% (3 of 46).

### Post transplantation outcome

#### Biological outcome

**Fig. 1:**
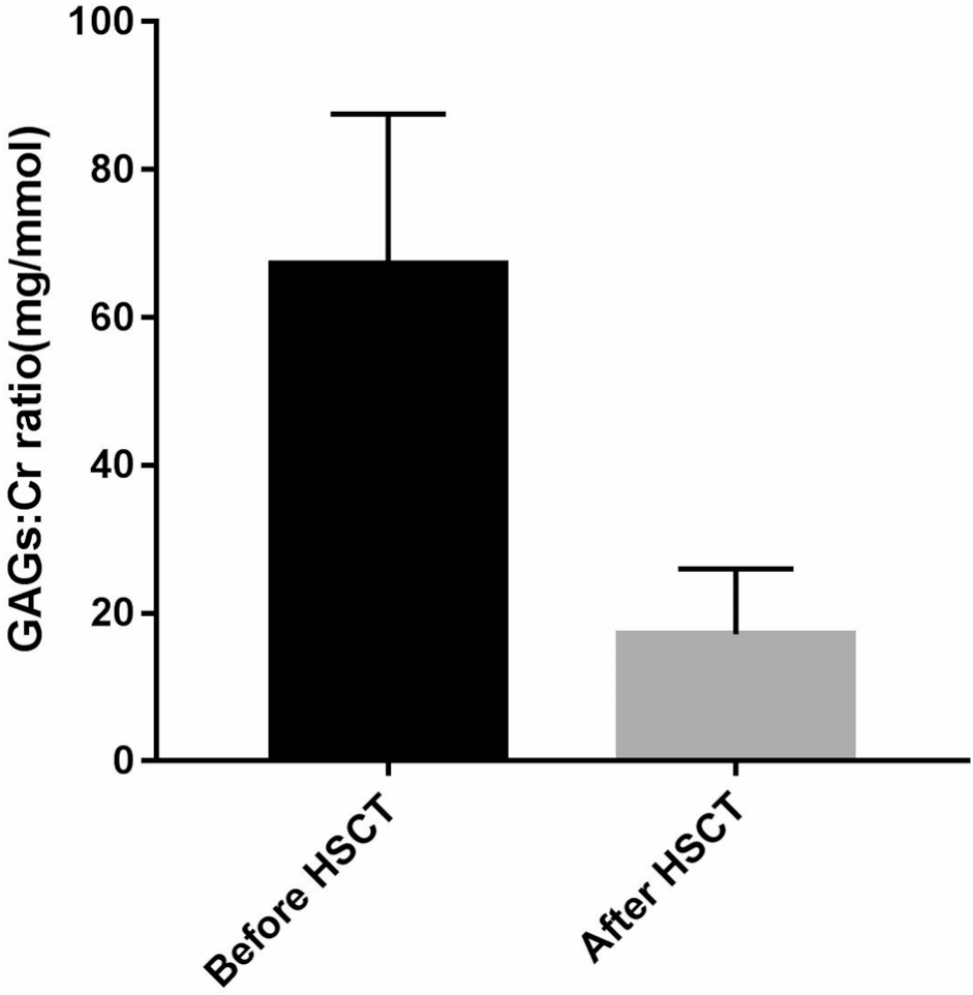
Urinary glycosaminoglycans: creatinine (GAGs: Cr) ratio recorded before and after HSCT

A total of 43 patients (93.5%, 43 of 46) achieved complete donor chimerism, three achieved mixed chimerism and one died early (thus, the status of chimerism was not assessed). The median time to neutrophil engraftment was 21 days (range 11–31 days). Among the 43 patients with successful engraftment, GAGs urinary excretion decreased to the upper limit of age-matched control values (Fig. 1) and enzyme activity levels reached normal levels.

### Clinical outcome

#### Growth

After HSCT, the patients with MPS IH/S and MPS II reached a nearly normal growth rate for height and weight. Meanwhile, the patients with MPS IH, MPS IVA and MPS VI remained very short and had poor growth after HSCT, especially for MPS IVA. For MPS IH/S, MPS IH, MPS II, MPS IVA and MPS VI, the growth rate of height and weight were 5.13 ± 0.61 cm, 3.62 ± 0.47 cm, 5.4 ± 0.51 cm, 1.79 ± 0.27 cm, 3.38 ± 0.35 cm and 1.52 ± 0.44 kg, 1.28 ± 0.36 kg, 1.67 ± 0.23 kg, 0.98 ± 0.22 kg, 0.77 ± 0.16 kg per year respectively. The average standard deviation scores (SDS) of height and weight after HSCT were − 0.2.52, -4.16, -0.65, -6.03, -3.51 and − 1.43, -2.36, -0.12, -2.84, -2.91 respectively for MPS IH/S, MPS IH, MPS II, MPS IVA and MPS VI.

#### Multiple bone dysplasia

All 43 patients presented with typical coarse facial features and stiffness of joints. These were improved or remained stable in most patients after HSCT (Table 2).

**Table 2:** Clinical outcomes in patients with MPS after transplantation (*n* = 43)

Clinical Outcome	Number of cases After HSCT				
	total	MPS I	MPS II	MPS IVA	MPS VI
**Coarse faces**	43	8	15	14	6
Improved	31	7	15	6	3
Stable	10	1	0	6	3
Worsened	2	0	0	2	0
**Stiffness of joints**	43	8	15	14	6
Improved	33	8	12	7	6
Stable	9	0	3	6	0
Worsened	1	0	0	1	0
**Thoracic and spinal deformities**	40	7	13	14	6
Improved	17	4	7	3	3
Stable	13	3	5	2	3
Worsened	10	0	1	9	0
**Hip dysplasia**	39	6	13	14	6
Improved	15	5	6	2	2
Stable	17	1	7	5	4
Worsened	7	0	0	7	0
**Genu valgum**	37	8	9	14	6
Improved	15	6	8	1	0
Stable	11	2	1	2	6
Worsened	11	0	0	11	0
**Upper-airway obstruction**	34	7	15	10	2
Improved	33	7	15	9	2
Stable	0	0	0	0	0
Worsened	1	0	0	1	0
**Hearing impairment**	21	4	9	6	2
Improved	11	4	5	1	1
Stable	10	0	4	5	1
Worsened	0	0	0	0	0
**Recurrent otitis media**	22	4	10	5	3
Improved	17	4	8	2	3
Stable	5	0	2	3	0
Worsened	0	0	0	0	0
**Decreased vision**	20	7	0	9	4
Improved	2	2	0	0	0
Stable	14	4	0	6	4
Worsened	4	1	0	3	0
**Corneal clouding**	21	7	0	8	6
Improved	12	6	0	0	6
Stable	7	0	0	7	0
Worsened	2	1	0	1	0
**Valvular heart disease**	27	7	12	3	5
Improved	4	0	4	0	0
Stable	23	7	8	3	5
Worsened	0	0	0	0	0
**Motor retardation**	24	5	4	13	2
Improved	14	5	3	4	2
Stable	8	0	0	8	0
Worsened	2	0	1	1	0
**Speech impairment**	17	4	10	0	3
Improved	9	3	3	0	3
Stable	7	0	7	0	0
Worsened	1	1	0	0	0
**cognitive function**	14	2	12	0	0
Improved	6	0	6	0	0
Stable	4	1	4	0	0
Worsened	3	1	2	0	0
**Hepatosplenomegaly**	27	8	15	3	1
Improved	20	6	10	3	1
Stable	7	2	5	0	0
Worsened	0	0	0	0	0
**Hydrocephalus**	6	1	4	1	0
Improved	2	0	2	0	0
Stable	3	0	2	1	0
Worsened	0	0	0	0	0

Thoracic and spinal deformities, hip dysplasia and genu valgum were improved or remained stable in most patients with MPS I, MPS II and MPS VI. However, these features worsened in more than half of the MPS IVA patients (Table 2).

#### Ear, nose and throat (ENT) symptoms

Upper-airway obstruction and recurrent otitis media were improved significantly in most patients with MPS after HSCT. Hearing impairment remained stable in patients with MPS after HSCT (Table 2).

#### Vision

Twenty patients had normal vision and the others had impaired vision; all patients with MPS II had normal vision; it was improved in 2 patients, stabilized in 14 patients and worsened in 4 patients after HSCT. Before HSCT, 21 patients had visible corneal clouding and none were among the patients with MPS II; following HSCT improvement was seen in 11 patients (Table 2). No cataracts were observed.

#### Cardiovascular signs and symptoms

Valvular involvement was present in 27 patients. It was improved in 4 patients, stabilized in 23 patients after HSCT and none worsened after HSCT (Table 2).

#### Neurological outcome

The motor retardation was improved in most patients with MPS after HSCT. The impairment of speech was improved in most patients with MPS I and MPS VI after HSCT; it remained stable in most patients with MPS II (Table 2).

The deterioration of cognitive function was found in 2 patients with MPS I, 12 patients with MPS II and none were among the patients with MPS IVA and MPS VI before HSCT. It was improved in 6 patients, stabilized in 5 patients after HSCT and 3 worsened after HSCT (Table 2).

#### Hepatosplenomegaly

Hepatosplenomegaly was present in 30 patients. It was improved in 21 patients, stabilized in 9 patients after HSCT and none worsened after HSCT (Table 2).

#### Hydrocephalus

Hydrocephalus was noted in 8 patients. It was improved in 5 patients, stabilized in 2 patients after HSCT and none worsened after HSCT (Table 2).

#### ADL scores

**Fig. 2:**
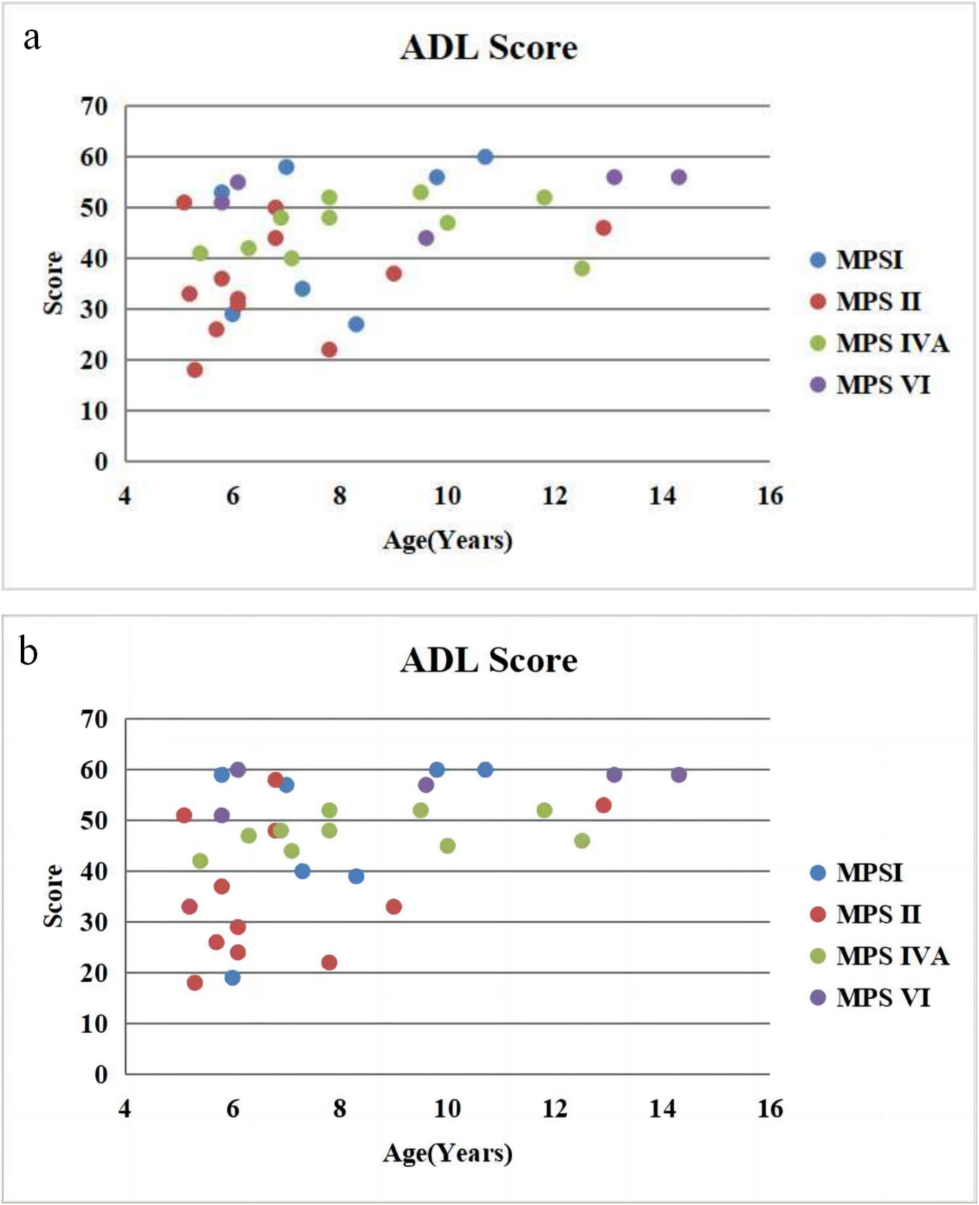
ADL score for patients with MPS before^a^ and after^b^ HSCT

**Fig. 3:**
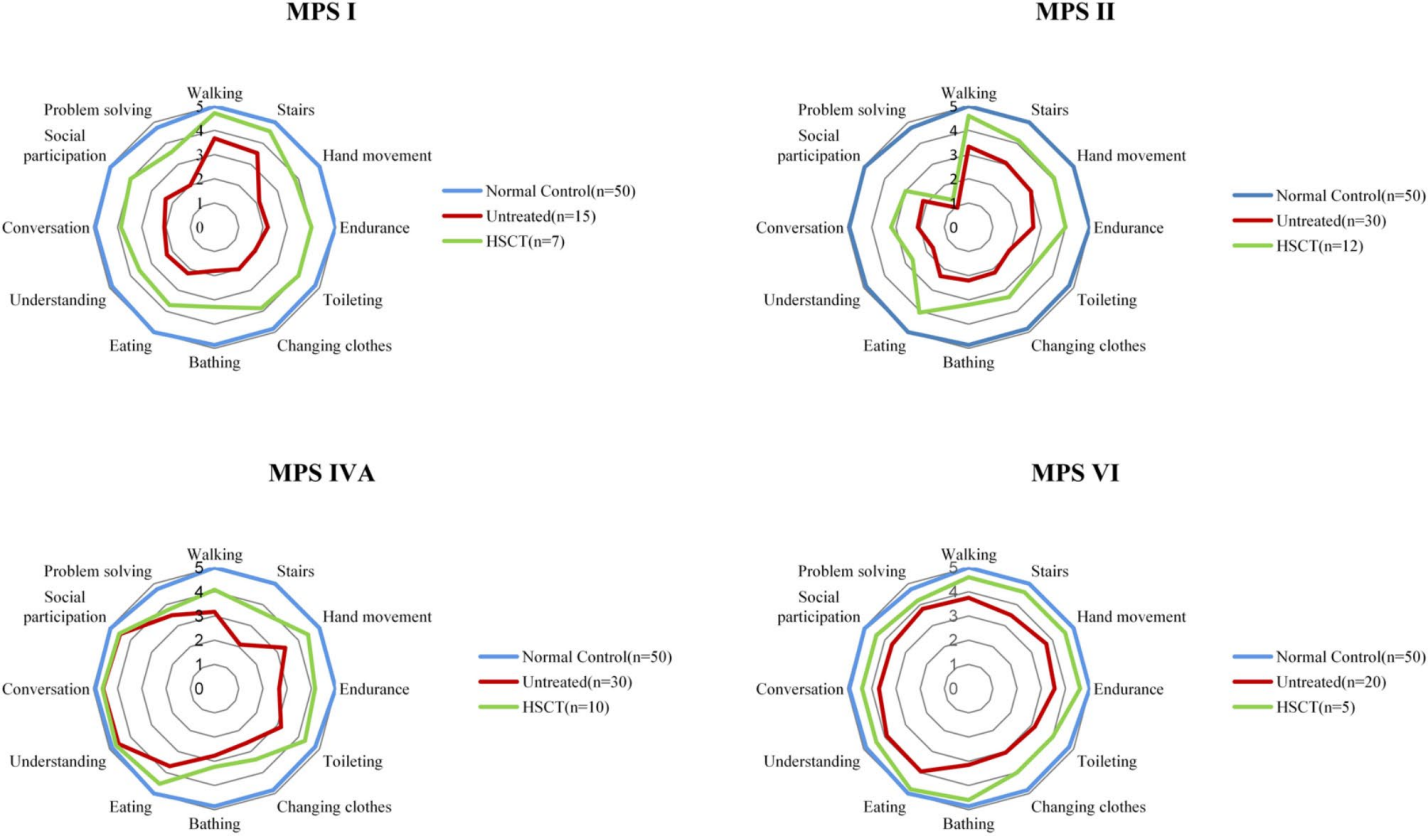
ADL scores in patients with MPS with or without HSCT. The graph shows the mean of the scores for each item. All subjects are over 5 years of age

Thirty-four patients completed a range of questionnaires after HSCT (Fig. 2). In 7 patients with MPS І, 4 were above 55 with MPS ІS, 2 were about 40 with MPS ІH/S and 1 was below 20 with MPS ІH (Fig. 3). In 12 patients with MPS II, 4 were above 40 with attenuated type and 8 were below 40 with severe type (Fig. 3). All the 10 patients with MPS IVA were above 44 (Fig. 3). All the 5 patients with MPS VI were about 50 (Fig. 3).

The overall average score in patients with MPS I after HSCT was 47.7 ± 15.5 (*n* = 7), far better than untreated patients (mean 28.1 ± 5.6, *n* = 15, *P* < 0.05) and less than normal controls (mean 58.9 ± 0.1, *n* = 50, *P* < 0.05). The overall average score in patients with MPS II after HSCT was 36.0 ± 13.3 (*n* = 12), far better than untreated patients (mean 27.6 ± 5.3, *n* = 30, *P* < 0.05) and less than normal controls (mean 58.9 ± 0.1, *n* = 50, *P* < 0.05). The overall average score in patients with MPS IVA after HSCT was 47.6 ± 3.5 (*n* = 10), far better than untreated patients (mean 37.5 ± 5.7, *n* = 30, *P* < 0.05) and less than normal controls (mean 58.9 ± 0.1, *n* = 50, *P* < 0.05 ). The overall average score in patients with MPS VI after HSCT was 57.2 ± 3.6 (*n* = 5), far better than untreated patients (mean 42.8 ± 4.9, *n* = 20, *P* < 0.05) and no different from normal controls (mean 58.9 ± 0.1, *n* = 50, *P* > 0.05).

## Discussion

HSCT is considered as an effective treatment option for patients with MPS I, II, IVA, VI, and VII. HSCT replaces endogenous hematopoietic lineage cells with exogenous cells transplanted from a healthy donor [15, 16]. Stem cells isolated from a different person or umbilical cord blood units are used for HSCT. We followed the donor type hierarchy: noncarrier matched sibling, matched UCB and fully matched unrelated donor [17]. In our protocol, UCB have been used with increasing frequency. UCB is the optimal graft source for the transplantation of patients with MPS [18, 19]. Without full matching between the donor and recipient, a UCB graft significantly increases the possibility of finding a suitable unrelated donor [20]. UCB can also lower rates of acute and chronic GVHD and reduce the likelihood of viral infections [20]. Thus, in our study UCB was added to minimize the risk of GVHD when mismatched unrelated donors were selected. It has been speculated that the cell population in UCB can expand and differentiate into cell types such as osteoblasts, chondroblasts and neurons [20].

A busulfan-based myeloablative regimen was applied as the conditioning regimen. Busulfan + cyclophosphamide + thymoglobulin was the conditioning regimen used for PBSC transplantation, while fludarabine was added to the regimen for CB transplantation. The recent recommendation for successful engraftment has opted for the combination of busulfan-cyclophosphamide with the addition of thymoglobulins in unrelated transplants [20]. Our high rate of full-donor chimerism proved the effectiveness of these conditioning regimens.

This study reports a median 3.1 years (range, 0.8–8.1 years) follow-up in 46 patients with MPS I, MPS II, MPS IVA and MPS VI who underwent HSCT from matched or mismatched donors, either related or unrelated, in a single pediatric centre. There were 12 related and 34 unrelated transplants but 14 cases of HLA mismatches were present in the unrelated transplant group. Unrelated transplant outcome prove to be positive despite the incomplete HLA matching. Thus, without a matched sibling donor our strategy is to look for an unrelated donor. This is the preferred alternatives such as CB transplantation or haplo-identical transplantation. Out of our cohort of patients, 14 transplants were performed with HLA disparities between recipient and donor; 50% of unrelated transplants and 50% of related transplants being mismatched. Seven patients received haplo-identical HSCT from mismatched related donors combined with UCB. UCB was infused one day earlier than PBSCs. In most studies, haplo-identical HSCT is limited by poor immune reconstitution and/or a high incidence of GVHD recurrence [21]. UCB may provide more robust immune reconstitution and reduce the rates of GVHD recurrence, but the limited numbers of progenitor cells in the UCB unit result in slow and unpredictable platelet and neutrophil recovery [22].

Most patients who received transplantation achieved complete donor chimerism and a good survival rate (93.5%). Three patients who died did not achieve complete donor chimerism, including 1 case of MPS І, 1 case of MPS II and 1 case of MPS IVA. The extent of HLA matching did not affect the risk of transplantation-related complications, such as severe GVHD. It may be due to improvements in transplantation techniques and the early prevention and treatment of GVHD. There was no significant difference in survival among patients with different types of MPS. No significant difference was found between donor or graft source and survival rate. Among the 43 patients with successful engraftment, GAGs urinary excretion decreased to the upper limit of age-matched control values, and enzyme activity reached normal levels.

A post-transplantation follow-up was performed. The follow-up time was median 3.1 years (range, 0.8–8.1 years). A longer-term follow-up was needed for organ function assessments. After HSCT, the patients with MPS IH/S and MPS II reached nearly normal growth rates for height and weight. Meanwhile, the patients with MPS IH, MPS IVA and MPS VI remained very short and had poor growth after HSCT, especially for MPS IVA.

In different types of MPS, multiple bone dysplasia including typical coarse facial features, stiffness of joints, thoracic and spinal deformities, hip dysplasia, genu valgum and vision were improved and remained stable. Upper-airway obstruction and recurrent otitis media were improved significantly. Hearing impairment remained stable. Cardiovascular problem seemed to stabilize after an initial worsening. The motor retardation was improved. The impairment of speech was improved in MPS I and MPS VI, and it remained stable in MPS II. The deterioration of cognitive function was improved in 6 patients, stabilized in 5 patients after HSCT and worse in 3 patients after HSCT. The three patients whose cognitive function worsened after HSCT included 1 patient with MPS I and 2 patients with MPS II. Their deterioration of cognitive function was obvious before HSCT. Most cases of hepatosplenomegaly resolved within several months after HSCT. Hydrocephalus was noted in 8 patients and all the patients improved or stabilized after HSCT.

Currently HSCT is widely used to treat patients with MPS IH [23], but few studies have reported HSCT outcomes in patients with other types of MPS. The first successful allogeneic HSCT procedure for MPS I was performed in 1980, involving a 1-year-old boy with severe MPS I [24]. HSCT is considered the gold standard for treatment of severe MPS I (MPS IH). It can alleviate a number of disease symptoms and prolong the patient’s life span, especially when performed before the age of 2 years and prior to cognitive impairment [25, 26]. In this study there were 6 cases of MPS IH and 3 cases of intermediate MPS I (MPS IH/S). The median age at transplantation was 4.10y (range 9 m-8y11m). Except for one deaths, all successfully transplanted MPS I patients surviving at least 1 year 5 months after HSCT are alive to date, with a median age of 7.51y (range 2y4m-11y1m) at the last follow-up evaluation. Most patients with MPS I had improved disease symptoms after HSCT. One patient with MPS IH had obvious deterioration of cognitive function and received HSCT at the age of 3y4m, after HSCT his cognition function worsened.

In Japan, China, and Brazil, HSCT is the preferred treatment option for MPS II [9, 27]. Kubaski et al. collected data for 146 HSCT patients with MPS II including 27 new cases and 119 published cases [28]. The 146 HSCT cases were compared with 51 ERT and 15 untreated cases [28]. The study showed that most HSCT patients had greater improvement in somatic features, joint movements and ADL compared to ERT patient [28]. HSCT patients showed either improvement or no deterioration of neurological symptoms while abnormal manifestations became more extensive after ERT [28]. HSCT appeared to be more effective than ERT for MPS II in a wide range of disease manifestations which could be considered as a therapeutic option for this condition [28]. In the study of Wang et al., 12 MPS II patients with 2 to 6 years of age showed some improvements in motor and speech skills, suggesting that HSCT was also beneficial in improving intellectual development [10]. In this study there were 11 cases of severe MPS II and 5 cases of mild MPS II. The median age at transplantation was 3.89y (range 1y1m-6y6m). Except for one death, all successfully transplanted MPS II patients surviving at least 10 months after HSCT are alive to date, with a median age of 6.72 years (range 3y10m-13y4m) at the last follow-up evaluation. The deterioration of cognitive function in ten patients with MPS II was improved or stabilized after HSCT. Two patients with MPS II had obvious deterioration of cognitive function and received HSCT at the age of 4y9m and 3y2m individually, after HSCT their cognition function worsened. Growth in height in patients with MPS II tends to normal until 8 years of age after which growth markedly dicreases [29]. The current age of most patients with MPS II in this study was below 8 years of age. A longer-term follow-up of growth in height was needed to be evaluated.

Among the three patients whose cognition function worsened after HSCT, they were all diagnosed with severe type of MPS I/II according their phenotype and genotype. Before HSCT, they have showed development delay in motion and speech. It is suggested that the absence of developmental deterioration may be associated with a better neurocognitive outcome. HSCT may not effectively improve brain involvement of MPS I/II if developmental delays are already present before transplantation. It is possibly because donor cells cannot reach deep brain tissue [9]. The effect of HSCT on neurological involvement may be limited. For severe type of MPS I/II, the age for HSCT should be set low before 2.5 years of age or even younger.

Evidence supporting the use of HSCT in patients with MPS IVA is limited. The first successful case report of HSCT in MPS IVA was published in 2014 [30]. In 2016, Yabe et al. reported allogeneic HSCT cases in 4 patients with MPS IVA [31]. In 2016, Wang et al. reported the other 4 patients with MPS IVA treated with HSCT in China [10]. Overall, the long-term follow-up of HSCT in MPS IVA patients showed that this therapy achieved the donor’s GALNS activity level, improved pulmonary function, BMD and ADL, and reduced the frequency of surgical intervention, suggesting that HSCT could be a useful supportive treatment option for patients with MPS IVA [10, 30, 32]. In this study there were 15 cases of severe MPS IVA. The median age at transplantation was 3.61y (range 1y6m-7y4m). Except for one death, all successfully transplanted MPS IVA patients surviving at least 1 year after HSCT are alive to date, with a median age of 7.64 years (range 2y8m-12y11m) at the last follow-up evaluation. Overall, the follow-up of HSCT in MPS IVA patients showed that this therapy achieved normal GALNS activity level, improved multiple bone dysplasia and ADL. Meanwhile, HSCT has a limited impact on bone growth of MPS IVA since the growth of height and weight were significantly delayed after HSCT. Thoracic and spinal deformities, hip dysplasia and genu valgum worsened in more than half of patients with MPS IVA after HSCT. Patients with MPS IVA often require surgical interventions even after a successful engraftment. However, Yabe H.et al. reported that in the post-HSCT group surgical intervention was not observed in groups with 0–15 years and after 20 years of age. HSCT group had a lower surgical frequency compared with the untreated group [14].

Evidence supporting the use of HSCT in patients with MPS VI is currently lacking. A small number of case studies suggests that HSCT increases the enzymatic activity, and has a positive effect on joint mobility, ENT and cardiac manifestations, movement, ADL, as well as reducing facial dysmorphism [10, 33, 34]. Akyol et al. reported on 6 cases of severe MPS VI [32]. The median age at transplantation was 3.50y (range 1y2m-6y8m). All successfully transplanted MPS VI patients surviving at least 1 year after HSCT are alive to date, with a median age of 9.08 years (range 2y9m-14y4m) at the last follow-up evaluation [33]. In this study, the follow-up of HSCT in MPS VI patients showed that this therapy achieved the normal ARSB activity level, improved multiple bone dysplasia and ADL.

Beside HSCT and ERT, it has been proposed that the pharmacological chaperones was used in the treatment of MPS. They can restore the folding, trafficking, and biological activity of mutated enzymes. These molecules have been applied for MPS II, IVA, and IVB, showing a mutation-dependent enhancement of the mutated enzymes [35]. Since neither HSCT nor ERT can be effective in treatment of neurological symptoms of MPS, One of potential therapeutic method is substrate reduction therapy (SRT). However, chaperone and SRT remain a potential therapeutic approach, but currently, no effective medication is known to exist [36].

Gene therapy has been explored as a potential therapeutic for MPS. Gene therapy for MPS I was first attempted by groups using retroviral vectors to deliver the IDUA gene [37]0.1 This mechanism can be used to introduce a corrective gene into the genome of the patient to produce a widespread therapeutic effect in the body [37]. In Gentner et al.’s study, the children with MPS I received autologous hematopoietic stem and progenitor cells (HSPCs) transduced ex vivo with an α-L-iduronidase (IDUA)–encoding lentiviral vector after myeloablative conditioning [38]. All the patients showed prompt and sustained engraftment of genecorrected cells and had supraphysiologic blood IDUA activity within a month [38]. Pabinafusp alfa consists of human iduronate-2-sulfatase (IDS), the enzyme in which patients with MPS-II are deficient (Hunter syndrome), fused to the C-terminus of the heavy chain of an anti-human transferrin receptor (hTfR) antibody [39]. Its successful delivery across the blood brain barrier (BBB) into the CNS by way of TfR-mediated transcytosis has been demonstrated in animal models, along with the resultant effects of decreasing heparan sulfate (HS) accumulations in the brain [39]. Its regulatory approval for general use was approved in Japan in March 2021 as the first BBB-crossing ERT [39].

Although there is useful information revealed by this study, there are also limitations. First of all a long-time follow-up is needed. Considering the heterogeneity of our patients, different types of MPS, severity of disease and age of transplantation, we cannot draw a conclusion on which patient may have major or minor benefits from HSCT. It is true that the risks of HSCT have decreased with years, but still, if we find that there are group of patients, who do not benefit of this procedure, it would be wise to avoid the risk.

## Conclusion

In Summary, HSCT is a good therapeutic option for MPS I, II, IVA and VI, and improves the patients quality of life. With the availability of different donor sources and modification of the conditioning regimens the success rate of HSCT for MPS is encouraging. HSCT is considered the standard of care for patients with MPS I. For the patients with MPS II, HSCT could be considered as the preferred treatment option. Our study also provides powerful evidence that HSCT is an effective option for MPS IVA and VI. Limitations include having a small sample size for each subtype of MPS patients with HSCT and the limited period of follow-up. It makes evaluating the effect of treatment and comparing treatments difficult. Other promising strategies developed in recent years including gene therapy specifically for more effectively targeting treatment to the central nervous system.

## Electronic supplementary material

Below is the link to the electronic supplementary material.

Supplementary Material 1

## Data Availability

Relevant data generated or analyzed during this study are included in this published article. The other datasets used and/or analyzed during the current study are available from the corresponding author on reasonable request.

## Declarations

The study was carried out according to the amended Declaration of Helsinki.

## Abbreviations

HSCT: Hematopoietic stem cell transplantation
MPS: Mucopolysaccharidoses
ADL: Activities of Daily Living
GAG: Glycosaminoglycan
ERT: Enzyme replacement therapy
CB: Cord blood
PBSC: Peripheral blood stem cell
UCB: Unrelated cord blood
ATG: Thymoglobulin
CsA: Cyclosporin
MTX: Methotrexate
MMF: Mycophenolate Mofetil
ANC: Absolute neutrophil count
STR: Short tandem repeats
