# Supplementary material for: Allogeneic hematopoietic stem cell transplantation for mucopolysaccharidosis patients: a single-center experience and assessment of quality of life
Source: Ital J Pediatr. 2025 Mar 18;51:83. doi: 10.1186/s13052-025-01919-7 (PMC11921649; doi:10.1186/s13052-025-01919-7)
Supplement: Supplementary file 1 — Supplementary Material 1 [file 13052_2025_1919_MOESM1_ESM.doc]

Cover Letter

April. 12, 2024

Dear Editor-in-Chief,

We submit our manuscript entitled “Allogenic hematopoietic stem cell transplantation for mucopolysaccharidosis patients: A single-centre experience and assessment of quality of life” to Italian Journal of Pediatrics for publication. In our study, we investigate the efficacy and improvements in the quality of life of HSCT in pediatric patients with MPS I, II, IVA, and VI. A retrospective analysis of transplantation data from 46 cases of MPS from a single center in China, treated between December 2013 to March 2021, was conducted. HSCT is a good therapeutic option for MPS I, II, IVA, VI and improve quality of life of patients. HSCT is considered the standard of care for patients with MPS I. For the patients with MPS II, HSCT could be considered as the preferred treatment option. Our study also provides powerful evidence that HSCT is an effective option for MPS IVA and VI.

We wish our manuscript will be reviewed by the experts of this field. This paper is new. Neither the entire paper nor any part of its content has been published or has been accepted elsewhere. It is not being submitted to any other journal.

We believe the paper may be of particular interest to the readers of your journal.

Correspondence and phone calls about the paper should be directed to Li Liu at the following address，phone and fax number，and e-mail address.

Li Liu

Institute: Department of Genetics and Endocrinology, Guangzhou Women and Children’s Medical Center.

Address: 9 Jinsui Street, Guangzhou, Guangdong, 510623, China.

Tel: +862038076125

Fax: +862038076124

E-mail: [liliuchina@qq.com](mailto:xxhuang321@163.com).

Thanks very much for your attention to our paper．

Sincerely yours，

Li Liu
